# Knowledge, Awareness, and Perception of Dental Students Regarding Digital Dentistry in Egypt: A Cross-Sectional Study

**DOI:** 10.7759/cureus.71061

**Published:** 2024-10-08

**Authors:** Mohamed A Hall, Ahmed Z Mahmoud, Osama S Mohamed, Inas Karawia

**Affiliations:** 1 Alexandria Dental Research Center, Ministry of Health and Population, Alexandria, EGY; 2 Department of Prosthodontics, College of Dentistry, Arab Academy for Science, Technology, and Maritime Transport, El-Alamein, EGY; 3 Department of Dental Prostheses Manufacture Technology, Pharos University in Alexandria, Alexandria, EGY; 4 Department of Pediatric and Community Dentistry, Pharos University in Alexandria, Alexandria, EGY

**Keywords:** awareness, dental students, digital dentistry, egypt, knowledge, perception

## Abstract

Introduction

Dental students are the future of the dental profession, and since digital dentistry is becoming more prevalent, they must receive an education that incorporates digital workflows and practices. This study aimed to determine the level of dental students' knowledge, awareness, and perception (KAP) of digital dentistry, as well as the influence of the type of school funding and information source on these aspects.

Methods

A cross-sectional online survey was performed between September 2023 and March 2024. Dental students from Egyptian public and private universities constituted our study sample. A 22-item questionnaire with four sections was employed for data collection. The data were analyzed using IBM SPSS Statistics for Windows, Version 25, (Released 2017; IBM Corp., Armonk, NY, USA).

Results

Out of 390 responses, 213 (54.6%) were females, 218 (55.9%) were students, and 287 (73.6%) received computer-aided design/computer-aided manufacturing (CAD/CAM) training in their dental schools. Most of the participants had a moderate to high level of knowledge (48.50% and 43.60%, respectively); 73.10% showed a moderate level of awareness; and 73.60% had a high level of perception of digital dentistry. Additionally, it was shown that interns and participants who gained their knowledge from social media and attended hands-on workshops had significantly higher KAP scores (p ≤ 0.05). The knowledge and perception scores were also impacted by attending a private dental school (p ≤ 0.05). Furthermore, reading books or articles considerably raised participants' awareness, whereas sharing knowledge with peers greatly enhanced their knowledge (p ≤ 0.05).

Conclusion

Our study revealed a moderate to high level of knowledge, a moderate level of awareness, and a high level of perception about digital dentistry among the majority of participants. Moreover, it underscores the need for comprehensive digital dentistry educational courses, emphasizing practical hands-on training - especially in public dental schools - to equip future dentists with the necessary knowledge and skills.

## Introduction

Digital dentistry has revolutionized various aspects of modern dentistry. The use of computer-aided design/computer-aided manufacturing (CAD/CAM) technology offers numerous benefits over conventional methods. These benefits include improved speed and simplicity, superior restoration quality, fewer steps required, time savings, and an easier procedure compared to traditional methods involved in creating a prosthesis [1]. It utilizes digital technologies to enhance dental procedures and treatments, aiming to improve efficiency, precision, and patient satisfaction. Furthermore, it has various applications, such as digital imaging, milling, 3D printing, intraoral scanning, virtual treatment planning, and artificial intelligence-driven digital diagnosis. These tools revolutionize dental practice by streamlining processes and offering advanced diagnostic and treatment capabilities [2,3].

Every dental school should consider curriculum change to be an integral part of its fundamental educational role. The investment in curriculum development is crucial for optimizing the educational journey of each student and ensuring that graduates are equipped to practice dentistry effectively, efficiently, and with compassion in a world undergoing continuous evolution in knowledge, technology, and cultural standards [4]. As dental students represent the profession's future, dental curricula must incorporate the most recent advanced equipment due to the prevalence of digital tools and applications in dental practice today. It is essential to establish digital education standards that are broadly adopted by various dental universities [5]. Consequently, the integration of digital technologies into dental curricula has commenced globally, with penetration levels varying based on local resources and demands, with the imperative need to constantly adapt and incorporate technological advancements into dental practice being one of the foremost challenges in digital education [5].

In order to evaluate the current state of theoretical and practical CAD/CAM instruction in undergraduate dental programs, a recent study was conducted in 2022 among 47 universities in the Middle East and North African (MENA) region. According to the study, 80% of the participating schools agreed that CAD/CAM should be mandatory for undergraduate students and should be encouraged. It is also apparent that short- or long-term training courses in this area are needed because the majority of participating dental colleges still did not include CAD/CAM in their undergraduate curricula [6]. While attempting to assess the students' opinions on the integration of CAD/CAM courses into dentistry curricula, Schlenz et al. conducted a survey, which found that the majority of the participants thought about treating patients in their dental clinics in the future using digital tools. Moreover, it demonstrated the favorable perspective of learners regarding the integration of digital dentistry into the preclinical curriculum. However, it was noted that some students had trouble using CAD/CAM systems [7].

In Egypt, five-year Bachelor of Dental Surgery (BDS) programs, followed by a year of internships, are available from both public and private colleges. The number of private dentistry schools has recently increased, and their tuition costs are significantly greater than those of public schools funded by the government [8]. The resources devoted to digital dentistry education, such as investments in modern technology, specialized training courses, and research opportunities, can be greatly impacted by the financial standing of dental schools [6]. While schools sponsored by private or corporate sponsors may have more financial flexibility to invest in cutting-edge technologies and educational initiatives, government-funded schools may face budgetary constraints that impact their ability to provide comprehensive training in digital dentistry. While a previous study that included dentists with different specialties and practicing in different institutions in Egypt revealed that most participants had moderate knowledge and awareness of digital dentistry, with dentists practicing in academia having the highest level of knowledge among their peers [9], to the best of the author's knowledge, no study has been done to evaluate undergraduate students' knowledge, awareness, or perception (KAP) of this technology.

This study aimed to address this knowledge gap by evaluating the level of KAP of digital dentistry among dental students in Egypt, as well as the influence of the type of school funding and information sources on these aspects.

## Materials and methods

Study design and setting

This study is a cross-sectional study based on an online survey using Google Form (Google, Inc., Mountain View, CA, USA). The study was conducted between September 2023 and March 2024, after the approval of the Research Ethics Committee at Pharos University (04-2022-11-27-3-047). The researcher followed the International Guidelines for Research Ethics and the World Medical Association Declaration of Helsinki (Version 2013). A cover letter at the beginning of the Google Form informed the participants of the study's purpose, its voluntary and anonymous nature, as well as the duration required to answer the questions. Before the questionnaire was filled out, written informed consent was obtained. The study complied with the Strengthening the Reporting of Observational Studies in Epidemiology (STROBE) reporting guidelines for observational studies.

Population and sampling method

The research included a group of dental students and intern dentists studying in Egypt's private or public faculties of dentistry. The inclusion criteria were undergraduate senior dental students and interns, irrespective of age and gender. The exclusion criteria included dental students in the pre-clinical years, uncompleted questionnaires, and participants in the pilot study. For this study, two sampling techniques were used. Convenience sampling was the first method used, in which members of the dental student union and interns in different faculties were directly emailed and messaged with the survey link. The second technique was snowball sampling, in which the students who filled out the questionnaire were instructed to send their peers the link via social media groups. The responses were set to allow only one response to prevent multiple entries.

Sample size

Since there was no previous literature addressing dental students’ knowledge regarding digital dentistry in Egypt, we assumed that 50% of the students had sufficient knowledge about digital dentistry [10] and calculated the minimum required sample size to be 378, with a precision of 5% at a 95% confidence level, using Epi-INFO Version 7.2 (Centers for Disease Control and Prevention (CDC), Atlanta, GA, USA). Our sample was structured to be reflective of an approximate population of about 21,526 senior dental students and interns at different Egyptian universities [11].

Data collection tool

A 22-item self-administered questionnaire in English (Appendix 1) was used (Table 6). The pre-validated questionnaire was adopted from previous literature, with some changes made to the demographic questions, and "I don't know" responses were added to the knowledge and awareness sections [12]. The content validity of the questionnaire was re-assessed by sending it to a panel of experts to ensure the quality of the data. Furthermore, it was pilot-tested on 20 participants to ensure the clarity of the questions. The reliability was checked, and the Cronbach’s alpha was 0.78. Demographic information (age, gender, type of dental school, degree of education, whether they received CAD/CAM training or not in dental school, and source of information on digital dentistry) was covered in the first part. The participants' knowledge was assessed with five questions in the second section. The third section, which included four questions, evaluated awareness; each correct response was scored one point, while incorrect responses, such as "I don't know," "None," and "No," were scored zero points. The study participants' perceptions and practices were examined in the fourth section, which included seven questions with a score of two points for "yes," one point for "no," and zero points for "not sure." Based on the following scores, the participants were evaluated.

Knowledge scores were categorized as follows: high (18-26), medium (9-17), and low (0-8). Awareness scores were classified into high (8-10), medium (4-7), and low (0-3). Perception scores were divided into high (10-14), medium (5-9), and low (0-4).

Study outcome

Our outcome was to assess the level of KAP of dental students and interns on the topic of digital dentistry, which was analyzed relative to their demographic data (age, gender, level of education, type of dental school, and source of information).

Statistical analysis

The collected data were revised, coded, and analyzed using IBM SPSS Statistics for Windows, Version 25 (Released 2017; IBM Corp., Armonk, NY, USA) for tabulation and analysis. The significance of the obtained results was judged at a 5% level. Categorical variables were presented by frequency and percent, and mean ± SD was used for numerical data presentation. Linear regression examined the association between participants' characteristics and KAP values. A one-way analysis of variance (ANOVA) test was used to compare more than two means.

## Results

Table 1 shows the distribution of the study participants according to their characteristics. It was found that 177 (45.4%) were males, 213 (54.6%) were females, 188 (48.2%) were in public dental schools, 218 (55.9%) were students, and 172 (44.1%) were intern dentists. Notably, 287 (73.6%) stated receiving theoretical or practical CAD/CAM training in dental school. Information on digital dentistry was sourced from conferences/webinars, workshops, social media, books, and colleagues. Participants' percentages varied, with 117 (34.7%), 88 (26.1%), 204 (60.5%), 58 (17.2%), and 131 (38.9%), respectively.

**Table 1:** Distribution of the study participants according to their characteristics CAD/CAM: Computer-aided design/computer-aided manufacturing

Variables	Total (n = 390)
Age, mean ± SD	22.8 ± 1.3
Gender, n (%)	
Male	177 (45.4)
Female	213 (54.6)
Dental school type, n (%)	
Public	188 (48.2)
Private	202 (51.8)
Level of education, n (%)	
Student	218 (55.9)
Intern dentist	172 (44.1)
Received any CAD/CAM training in dental school, n (%)	
Yes	287 (73.6)
No	103 (26.4)
Other sources of knowledge (multiple answers allowed), n (%)	
Conference/webinar	117 (34.7)
Hands-on workshops/course	88 (26.1%)
Social media	204 (60.5%)
Books/articles	58 (17.2%)
Colleagues	131 (38.9%)

**Figure 1:**
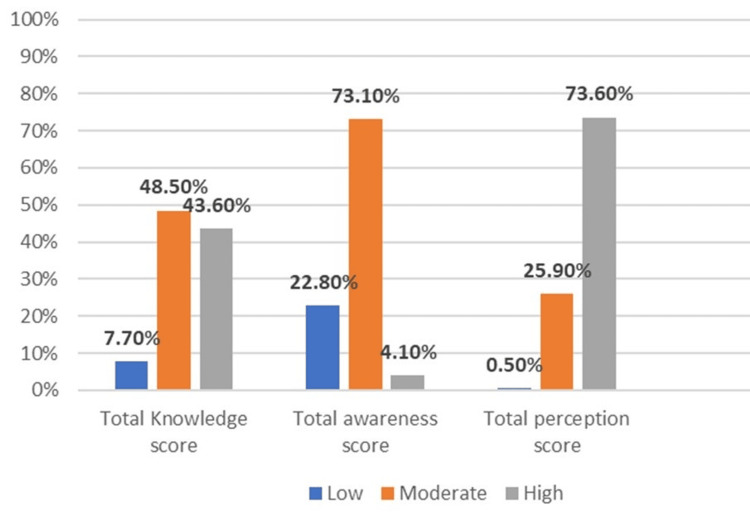
The total knowledge, awareness, and perception scores among participants

The study revealed that only 7.70% (30) of participants had low knowledge, while 48.50% (190) and 43.60% (170) had moderate and high knowledge, respectively. About 73.10% (285) had moderate awareness, while 22.80% (89) and 4.10% (16) had low and high awareness, respectively. Perception was low in 0.50% (2) of the participants, moderate in 25.90% (101), and high in 73.60% (287) (Figure 1).

In regression analysis (Table 2), it was found that being an intern dentist or affiliated with a private dental school significantly affects the knowledge level (B = 2.39, p = 0.000; B = 1.60, p = 0.006). The knowledge score was not significantly affected by gender or by whether the participants received CAD/CAM training in dental school (B = 0.743, p = 0.158; B = 0.097, p = 0.876). Regarding other sources of information, it was found that attending hands-on workshops/courses, gaining knowledge from social media, and sharing knowledge with colleagues had a highly significant effect on increasing knowledge (B = 2.27, p = 0.001; B = 2.34, p = 0.000; B = 1.32, p = 0.019). However, attending conferences/webinars or reading books/articles had no significant effect on total scores (B = 0.34, p = 0.572; B = 1.14, p = 0.125).

**Table 2:** Relationship between participants' characteristics and the knowledge of digital dentistry F-value = 7.6; p-value = 0.000; Adjusted R-square = 13.3% * indicates significance at p-value ≤ 0.05; Ref indicates the reference variable; - indicates empty cell CAD/CAM: Computer-aided design/computer-aided manufacturing

Variables		Knowledge		
		B	t	p-value
Gender	Male ^Ref^	-	-	-
	Female	0.743	1.4	0.158
Level of education	Student^ Ref^	-	-	-
	Intern dentist	2.39	4.2	0.000*
Dental school type	Public^ Ref^	-	-	-
	Private	1.60	2.75	0.006*
Receiving any CAD/CAM training in dental school	No^ Ref^	-	-	-
	Yes	0.097	0.16	0.876
Other source of knowledge				
Conferences/webinar	No^ Ref^	-	-	-
	Yes	0.34	0.57	0.572
Hands-on workshops/courses	No^ Ref^	-	-	-
	Yes	2.27	3.47	0.001*
Social media	No^ Ref^	-	-	-
	Yes	2.34	4.33	0.000*
Books/articles	No^ Ref^	-	-	-
	Yes	1.14	1.54	0.125
Colleagues	No^ Ref^	-	-	-
	Yes	1.32	2.36	0.019*

Table 3 shows that there were no significant differences in levels of awareness across genders, types of dental schools, and whether participants received CAD/CAM training (B = 0.257, p = 0.118; B = 0.326, p = 0.075; B = 0.369, p = 0.059, respectively). Being an intern dentist had a significant positive impact on the total awareness score (B = 0.728, p = 0.000). Regarding other sources of information, it was found that attending conferences or webinars and exchanging knowledge with colleagues had no significant impact on increasing participants' awareness (B = 0.126, p = 0.50; B = 0.049, p = 0.780). On the contrary, it was found that attending hands-on workshops or courses, searching on social media, and reading books or articles had a significant positive effect on increasing participants' awareness (B = 0.829, p = 0.000; B = 0.640, p = 0.000; B = 0.621, p = 0.008).

**Table 3:** Relationship between participants' characteristics and the awareness of digital dentistry F-value = 7.0; p-value = 0.000; adjusted R-square = 12.2% * indicates significance at p-value ≤ 0.05; Ref indicates the reference variable; - indicates empty cell CAD/CAM: Computer-aided design/computer-aided manufacturing

Variables		Awareness		
		B	t	p-value
Gender	Male ^Ref^	-	-	-
	Female	0.257	1.567	0.118
Level of education	Student^ Ref^	-	-	-
	Intern Dentist	0.728	4.106	0.000*
Dental school type	Public^ Ref^	-	-	-
	Private	0.326	1.785	0.075
Receiving any CAD/CAM training in dental school	No^ Ref^	-	-	-
	Yes	0.369	1.897	0.059
Other source of knowledge				
Conferences/webinar	No^ Ref^	-	-	-
	Yes	0.126	0.676	0.500
Hands-on workshops/courses	No^ Ref^	-	-	-
	Yes	0.829	4.042	0.000*
Social media	No^ Ref^	-	-	-
	Yes	0.640	3.782	0.000*
Books/articles	No^ Ref^	-	-	-
	Yes	0.621	2.678	0.008*
Colleagues	No^ Ref^	-	-	-
	Yes	0.049	0.280	0.780

It was found, as shown in Table 4, that neither gender (B = 0.035, p = 0.851) nor receiving any CAD/CAM training in dental school (B = 0.011, p = 0.959) had a significant impact on the total perception score. However, being an intern (B = 0.402, p = 0.046) or being affiliated with a private dental school (B = 1.053, p = 0.000) had a significant positive effect on the total perception score. Concerning other sources of information, it was found that attending hands-on workshops or courses (B = 0.553, p = 0.018) or searching on social media (B = 0.413, p = 0.032) had a significant positive impact on increasing the total perception score. On the other hand, attending conferences or webinars (B = 0.001, p = 0.997), reading books or articles (B = 0.141, p = 0.592), or exchanging knowledge with colleagues (B = 0.364, p = 0.067) had no significant effect on the total perception scores of the participants.

**Table 4:** Relationship between participants' characteristics and the perception of digital dentistry F-value = 5.6; p-value = 0.000; adjusted R square = 9.7% * indicates significance at p-value ≤ 0.05; Ref indicates the reference variable; - indicates empty cell

Independent variables		Perception		
		B	t	p-value
Gender	Male ^Ref^	-	-	-
	Female	0.035	0.187	0.851
Level of education	Student ^Ref^	-	-	-
	Intern dentist	0.402	1.99	0.046*
Dental school type	Public^ Ref^	-	-	-
	Private	1.053	5.09	0.000*
Receiving any CAD/CAM training in dental school	No^ Ref^	-	-	-
	Yes	0.011	0.052	0.959
Other source of knowledge				
Conferences/webinar	No^ Ref^	-	-	-
	Yes	0.001	0.003	0.997
Hands-on workshops/courses	No^ Ref^	-	-	-
	Yes	0.553	2.383	0.018*
Social media	No^ Ref^	-	-	-
	Yes	0.413	2.151	0.032*
Books/articles	No^ Ref^	-	-	-
	Yes	0.141	0.537	0.592
Colleagues	No^ Ref^	-	-	-
	Yes	0.364	1.838	0.067

When a comparison was made between the participants who obtained their knowledge from one, two, and three or more sources (Table 5), it was found that the mean knowledge gained from three or more sources (19.0 ± 4.8) was significantly higher than that of those who gained their knowledge from two sources (17.3 ± 4.4), which in turn was significantly higher than that of those who gained their knowledge from one source of information (15.8 ± 6.2) (p = 0.01). For total awareness scores, the means of awareness obtained from two sources (4.9 ± 1.6) or three or more sources (5.1 ± 1.4) were significantly higher than those obtained from one source of knowledge (4.3 ± 1.9) (p = 0.006). Concerning total perception, when students gained information from two sources (10.8 ± 1.7), it was not statistically significant compared to one source (10.4 ± 2.0) or three or more sources (11.3 ± 1.6). However, when comparing the mean perception of one source and three or more sources, there was a statistically significant difference (p = 0.008).

**Table 5:** Comparison between the number of sources on the total knowledge, awareness, and perception of participants * indicates significance at p-value ≤ 0.05 a, b, c: Different manuscript letters indicate significant differences between each pair

Number of sources	Total knowledge	Total awareness	Total perception
One source, n = 102	15.8 ± 6.2^a^	4.3 ± 1.9^a^	10.4 ± 2.0^a^
Two sources, n = 156	17.3 ± 4.4^b^	4.9 ± 1.6^b^	10.8 ± 1.7^ab^
Three or more sources, n = 132	19.0 ± 4.8^c^	5.1 ± 1.4^b^	11.3 ± 1.6^b^
p-value	0.01*	0.006*	0.008*

## Discussion

Digital dental technology has several positive implications for dentistry. Dental students, therefore, need to receive sufficient training, or, at the very least, be exposed to these tools and processes before graduating from dental school to be qualified to perform advanced dental treatments as technology develops and becomes increasingly integrated into dental practice. The purpose of this study was to address the gap in the literature by investigating dental students' KAP of digital dentistry in Egypt, as well as the impact of the information sources they rely on and the type of school on these aspects. The study's results showed significant variation based on the type of school that the participants attended, their level of education, and the sources of information they used.

Studies assessing dental students' KAP of CAD/CAM dentistry show a generally positive view among dental students. However, the students feel inadequately knowledgeable and need further instruction. Despite not being satisfied with their education, they consider CAD/CAM technology a significant part of dentistry and believe it will play an important role in the future [13-15]. This is in accordance with the results of the present study, where most participants had moderate knowledge and awareness levels while demonstrating a high level of perception regarding practicing digital dentistry. These findings highlight the potential need for a more comprehensive digital dental education, with greater emphasis on clinical training, to equip future dental professionals with a proper understanding of and practical skills related to this important topic.

The majority of Egypt's dental education was offered by governmental universities. However, private universities were authorized to begin offering BDS programs in the late 1990s, and ever since then, there have been more private dental schools, charging considerably higher tuition than public institutions that are funded by the government [8]. The results of this study showed that participants admitted to private dental schools had a significantly higher level of knowledge and perception than their peers in public schools. This may be attributed to the high costs of digital tools and, consequently, the greater availability of CAD-CAM units and digital equipment in private schools compared to public ones, which offers the students more exposure to and utilization of this equipment. This finding conflicts with research carried out in Pakistan [16] and Saudi Arabia [17], which showed no significant differences between dental schools based on the type of funding source they received.

Dental interns demonstrated a significantly higher degree of KAP in the current study. This is consistent with the results of the Alhamed et al. study in United States dental schools, which found that the longer a student attended dental school, the more they learned about the potential applications of this technology and the more subjective understanding of CAD/CAM they claimed to have [15]. Similarly, compared to their younger colleagues, interns in an Indian study reported having seen a CAD-CAM machine and had a slightly higher percentage of awareness regarding digital impressions, with significant differences existing between the groups [13]. Furthermore, Sheba et al.'s study found positive trends in digital dentistry knowledge scores, with second-year students showing lower scores and fourth-year students demonstrating higher intentions to use digital technology [18]. The consistency of the findings from this study and previous literature may be explained by the fact that interns’ exposure to current technologies is superior to that of their younger peers, and the aforementioned trend serves as a reliable predictor of how well students perform and how they grow throughout the course of their education.

Regarding the source of information that the participants relied on, the current study found that undergraduate education was not significantly associated with KAP scores, but rather predicted by social media, books, hands-on courses, and colleagues. On the contrary, a study at Zagreb's School of Dental Medicine found that students mostly learned about CAD/CAM technology through on-campus lectures without attending extra classes [14], which may reflect the differences in curriculum or educational techniques between the different countries.

In line with the findings of the present study, which revealed that participants who identified hands-on courses as their source of information were associated with significantly higher KAP scores, Schwindling et al.'s study found that a hands-on CAD/CAM module in a preclinical restorative dentistry course significantly outperformed a video-based lecture. Students also showed a high appreciation for the practical subject, and the study found that teaching digital restoration design in small groups through hands-on classes was beneficial, with the knowledge gained being worthwhile [19]. Furthermore, the current study's results showed that using social media for gaining knowledge was linked to noticeably higher KAP scores, which is consistent with earlier research that discovered that medical school final-year students utilized social media for their studies in addition to online learning tools like tutorials, case studies, videos, and quizzes [20]. This finding reflects the increases in internet accessibility and usability in recent years, which have made it possible for dentists and students to gain knowledge more rapidly and readily.

The current study found that consulting with colleagues increased knowledge, aligning with a multinational study involving dental students from Malaysia and Finland. On the contrary, reading books and articles were not popular among students from either country [21], which is inconsistent with the results of the present study and might be explained by differences in curriculum, educational techniques, or study tools available to students from different countries.

Participants who utilized multiple information sources showed higher KAP, highlighting the importance of accessing various sources in enhancing dental students' understanding of digital technology. Moreover, by engaging with a variety of sources, students gain greater depth and breadth of knowledge, which facilitates the synthesis of information, contributes to heightened awareness of the importance of modern technology in dental care, and influences their perceptions. These results underscore the importance of including a variety of information sources in dental educational programs in order to better prepare students for implementing contemporary technology in their practices.

Strength and limitations

There are a few limitations to this study. The design is cross-sectional and evaluates association rather than causality. Individuals who have a greater interest in technology may be more likely to respond to the online survey. Moreover, despite the anonymity of the questionnaire, social desirability might lead to response bias by encouraging participants to overestimate their knowledge. However, this is the first study that delves into dentistry students' knowledge of this crucial area. Students from Egypt's public and private dentistry schools, regardless of gender, have been included in the study sample. Future paper-based studies with a larger sample size are needed in order to confirm the results of the present study.

## Conclusions

The findings of the present study revealed that the majority of participants had a moderate to high level of knowledge, a moderate level of awareness, and a high level of perception about digital dentistry, which seemed to increase significantly with the number of sources they relied on to gain information. Furthermore, courses outside of dental school, social media, reading books, and consulting with colleagues were the main sources of information that participants used to gain knowledge. Due to the curiosity among students, which stems from the need to obtain information about digital dentistry - primarily sourced from outside the dental school - and considering that digital dentistry is becoming an integral part of future practice, it is recommended that dental schools adopt more hands-on module training for teaching digital dentistry, especially in governmental dental schools, to prepare dental students for blending into modern dental practice.

## Appendices

**Table 6:** Appendix 1

Question	Answers
Demographic section	
Q1) Gender	Male
	Female
Q2) Age	…….
Q3) You are	Dental student
	Intern
Q4) Which university do you belong to?	……..
Q5) Did you receive any theoretical or practical CAD-CAM training in dental school?	Yes
	No
Q6) What is your source of CAD-CAM knowledge? (multiple answers allowed)	Conferences/webinars
	Hands-on workshops/courses outside the dental school
	Social media
	Books/articles
	Colleagues
	Others
Knowledge section (please select all that apply)	
Q1) Which procedure in digital technology is useful in dentistry?	Crown and bridge fabrication
	Implant restorations
	Impression making
	Maxillofacial prosthesis
	Surgical reconstruction
	Smile designing
	Invisible orthodontics
	Other:
	I do not know
Q2) Use of CAD/CAM in dentistry	Intraoral scanning
	Digital impressions
	Shade matching
	Computer-aided designing (by laboratory or specialist milling center)
	Computer-aided manufacturing (by laboratory or specialist milling center)
	I do not know
Q3) Advantages of CAD/CAM	Reduced number of appointments
	Less chair side time
	More precise as compared to conventional methods
	Digital data flow
	Other: Specify:
	I do not know
Q4) Advantages of CAD/CAM in the clinical scenario	Eliminates the problems associated with impression-making
	Can review your preparation and modify it at the same time
	Immediate data transfer and retrievability of scan data at any point
	Ease in laboratory authorization and communication
	Accurate and precise fit of the restoration/orthodontic appliances
	Accurate and precise orthodontic tooth movement
	I do not know
Q5) Shortcomings of the use of CAD/CAM	High cost
	Lack of infrastructure
	Lack of knowledge
	Prefer conventional methods
	I do not know
Awareness section	
Q1) Are you aware of the CAD-CAM technology clinical applications in dentistry?	Yes
	No
Q2) Are you aware of the use of digital technology in dentistry?	Yes
	No
Q3) Which of the following CAD-CAM systems are you aware of? (please select all that apply)	Lava™
	Distributed Control System (DCS) Precident
	Chairside Economical Restoration of Esthetic Ceramic (CEREC)
	Procera
	None
Q4) Which of the following materials are used with CAD-CAM systems? (please select all that apply)	Emax
	Zirconia
	Metals
	Composite
	I do not know
Perception/practice section	
Q1) In your opinion do you think that Digital Dentistry would have a role to play in the COVID-19 scenario?	Yes
	No
	Not sure
Q2) Are you willing to attend any training program or workshop on CAD/CAM?	Yes
	No
Q3) Do you think there is a need to increase knowledge regarding digital dentistry during undergraduate/postgraduate courses?	Yes
	No
	Not sure
Q4) Are you interested in incorporating CAD/CAM into your regular workflow?	Yes
	No
	Not sure
Q5) Would you prefer CAD/CAM over conventional methods?	Yes
	No
	Not sure
Q6) Do you think digital dentistry would affect your clinical decision-making?	Yes
	No
	Not sure
Q7) Do you think digital dentistry would have a positive impact on our profession and would be the future of dental practice?	Yes
	No
	Not sure
